# Sleep Duration and Quality and Sensory Reactivity in School-Aged Children: The Spanish Cross-Sectional InProS Study

**DOI:** 10.3389/fped.2021.646011

**Published:** 2021-07-05

**Authors:** Paula Fernández-Pires, Desirée Valera-Gran, Miriam Hurtado-Pomares, Cristina Espinosa-Sempere, Alicia Sánchez-Pérez, Iris Juárez-Leal, María-Pilar Ruiz-Carbonell, Paula Peral-Gómez, Irene Campos-Sánchez, María-Teresa Pérez-Vázquez, Eva-María Navarrete-Muñoz

**Affiliations:** ^1^Department of Surgery and Pathology, Miguel Hernández University, Alicante, Spain; ^2^Occupational Therapy Research Group (InTeO, Investigación en Terapia Ocupacional), Miguel Hernández University, Alicante, Spain; ^3^Miguel Hernández University-Vice Rectorade of Institutional Relations, Elche, Spain; ^4^Centro de Desarrollo Infantil Sentits, Alicante, Spain

**Keywords:** sleep duration, sleep quality, sensory processing, short sensory profile, childhood, sensory reactivity

## Abstract

**Background:** The relationship between children's sleep and health has been widely examined; however, research focused on the link between sleep and sensory reactivity in children without medical conditions is relatively new and based on studies with small samples. Hence, we aimed at exploring the association between sleep duration and quality and prevalence of sensory reactivity in a population-based sample of children aged 3–7.

**Methods:** We examined data on 579 school-age children from the InProS project, a cross-sectional population-based study. Children's sleep duration was classified as <10 vs. ≥10 h/day, and sleep quality was measured using the Pediatric Sleep Questionnaire, defining poor quality sleep as a score of ≥0.33. The Short Sensory Profile (SSP) was used to classify children with or without sensory reactivity using the cut-off points proposed by W. Dunn for SSP total score and each SSP subscale. Prevalence ratios (PR) using Poisson multiple regression models with robust variance were estimated to examine main associations.

**Results:** Around a third (32.6%; *n* = 189) slept <10 h/day and 10.4% presented poor sleep quality. The prevalence of sensory reactivity was 29.5% for total SSP (<155), 11.4% for tactile sensitivity (<30), 15% for taste/smell sensitivity (<15), 22.5% for movement sensitivity (<13), 49.1% for under-responsive/seeks sensation (<27), 44.4% for auditory filtering (<23), 12.4% for low energy/weak (<26), and 25.4% for visual/auditory sensitivity (<19). Main findings indicated that poor sleep quality was significantly associated with a greater prevalence of sensory reactivity for SSP total score (PR = 1.27; IC 95%: 1.18; 1.38), tactile sensitivity (PR = 1.09, IC95%: 1.00–1.19), taste/smell sensitivity (PR = 1.18, IC95%: 1.08–1.30), under-responsive/seeks sensation (PR = 1.28, IC95%: 1.20–1.37), auditory filtering (PR = 1.31, IC95%: 1.23–1.39), low energy/weak (PR = 1.14, IC95%: 1.04–1.25) and audiovisual sensitivity (PR = 1.15, IC95%: 1.05–1.26) scores after adjusting for potential confounders.

**Conclusions:** In this study, we observed that poor sleep quality was statistically significantly associated with a higher prevalence of sensory reactivity as measured by the total SSP and almost all SSP subscales. To our knowledge, this is the first time that this association has been explored and reported. Further research from prospective studies is required to confirm these findings.

## Introduction

Sensory processing is the capacity of the central nervous system to process and give an adaptive response to environmental stimuli received from sensory systems (tactile, olfactory, gustatory, auditory, visual, proprioceptive, and vestibular) (1). However, when sensory processing does not trigger an efficient and/or appropriate response to registered stimuli, it could be indicative of the presence of sensory processing difficulties, including sensory sensitivity and/or sensory reactivity (2). Children with developmental problems such autism spectrum disorder (ASD) or attention deficit hyperactivity disorder (ADHD) are particularly vulnerable to sensory processing issues (1, 3), although, several previous studies reported that the prevalence of these problems may affect between 5 and 18 % of children from the general population (1, 3–6). Importantly, children suffering from such health problems are especially prone to experiencing significant difficulties that compromise their participation in social and family activities, school achievement, and/or development of their autonomy in the activities of daily life (4, 7–10). However, to the best of our knowledge, there is a general lack of understanding about sensory processing issues in typically developing children, or more specifically, which factors are associated with, or how sensory processing issues such as sensory reactivity can affect children's health and normal development.

Sleep is a complex and dynamic process regulated by the central nervous system through circadian rhythms and homeostasis that control sleep-wake needs (11). There are both exogenous and endogenous factors (e.g., light, social habits; or body temperature, hormone changes, respectively) that can affect sleep-wake cycle regulation (12–14). Although, the knowledge about the function and regulation of sleep is still limited, it is widely recognized that healthy sleep is crucial for good health and well-being (15) and includes achieving adequate sleep duration, good sleep quality, keeping appropriate sleep timing, and having no sleep disorders (16). Research on pediatric population indicates that sleep duration, timing, quality and variability are increasingly being associated with a wide range of health outcomes such as body composition, emotional regulation, growth, cognition, academic achievement, quality of life, or cardiometabolic outcomes (15, 17). Although, the relationship between children's sleep and health has been widely examined by a large body of literature (15, 17), as far as we know, research focused on the link between sleep and sensory reactivity in children without medical conditions is relatively new and based on study with small samples (18–23).

Briefly, Shochat and colleagues observed that specific sensory reactivity such as tactile sensitivity, movement sensitivity, auditory filtering as well as global sensory processing scores were negatively correlated with sleep in a study conducted with 56 children aged 8 in 2009 (20). More recently, several studies have suggested that sensory processing outcomes may be also negatively related to shorter sleep duration in children aged from 0 to 36 months (*n* = 177) (21) and to poor sleep habits in children from 6 months to 2.5 years of age (*n* = 160) (23) and in primary school children with ages between 7 and 8 and 12 (*n* = 45; *n* = 231) (19, 22). Consequently, in the light of the scant available results, however consistent, further, research is needed to provide more solid evidence about this health issue, most notably from large sample studies with high-quality epidemiological design. Hence, this study aimed at exploring the association between sleep (duration and quality) and sensory reactivity in a Spanish population-based sample of typically developing school-aged children.

## Materials and Methods

### Study Design and Population

The Infancia y Procesamiento Sensorial (InProS [Sensory Processing and Childhood], www.inteo.edu.umh.es/inpros) project is a cross-sectional population-based study carried out in typically developing children aged 3–7 in Alicante, Spain. Detailed information on the study protocol has been published previously (24). Briefly, around 1,700 eligible children from 21 schools randomly selected in Alicante province. They were invited to participate in this study through an envelope with an invitation letter addressed to their parents. After approximately a 2/3-week period and once all the documentation was examined, children were excluded from the study if they presented any disability. As such, children who had allergic conditions (*n* = 6), atopic dermatitis (*n* = 1), asthma (*n* = 1), bronchopulmonary dysplasia (*n* = 1), tumor (*n* = 1), ASD (*n* = 1), ADHD (*n* = 1) were excluded from the study analysis. Finally, a total sample of 620 children was included, rendering a response rate of about 37%. Recruitment of the study participants was performed between February and May of 2016. All children that finally participated in this study provided an informed consent signed by their parents. The Ethical Committee of the Miguel Hernandez University of Elche gave authorization to conduct this research project (DPC.ASP.02.16). For the present analysis, we examined data on 579 children (93.4%) with complete information for the main study variables.

### Study Variables

#### Sensory Reactivity

Child sensory processing was measured using the Short Sensory Profile (SSP) questionnaire validated and adapted for use in Spanish children population ranging in age from 3 to 10 (25). The SSP is an abbreviated version of the Sensory Profile created by W. Dunn (26) and specifically aimed at detecting sensory processing difficulties. This questionnaire is a parent report tool that measured child sensory reactions or behavioral responses to sensory stimuli and includes 38 items divided into seven different subscales: tactile sensitivity, taste/smell sensitivity, movement sensitivity, under-responsive/seeks sensation, auditory filtering, low energy/weak, and visual/auditory sensitivity. Each item can be rated on a Likert-type five-point scale ranging from 1 (al-ways) to 5 (never). The scores for total SSP and subscales can be derived by summing the responses of all respective values of the items. The scoring of the SSP (total and subscales) allows the classification of children into three different sensory processing profiles (typical performance, probable difference or definite difference) according to the cut-off criteria proposed by Dunn (26). Briefly, based on a large normative sample of children without disabilities (27), the criteria used for classifying children with “typical performance” were children who got total scores at or below one standard deviation from the mean. The criteria used for defining children with “probable difference” were those children that showed total scores higher than one standard deviation and <2 standard deviations from the mean. Finally, the criteria used for classifying children with “definite difference” were children who had scores >2 standard deviations from the mean. On the SSP, children were classified into “typical performance,” “probable difference,” and “definite difference” categories according to the total score for the total SSP and each SSP subscale, as follows, respectively: total SSP (≥155, 154–142, ≤141); tactile sensitivity (≥30, 29–27, ≤26); taste/smell sensitivity (≥15, 14–12, ≤11); movement sensitivity (≥15, 12–11, ≤10); under-responsive/seeks sensation (≥27, 26–24, ≤23); auditory filtering (≥23, 22–20, ≤19); low energy/weak (≥26, 25–24, ≤23); visual/audiovisual sensitivity (≥19, 18–16, ≤15). A complete summary of the scores for the total SSP and total SSP subscales and classification of the child sensory profile was previously published elsewhere (23). In our study, the children with sensory reactivity were defined as those classified into “probable difference” and “definite difference” categories of sensory profile that were later merged into only one category (i.e., sensory reactivity). As such, the total SSP and total SSP subscales were analyzed in dichotomous terms (no sensory reactivity vs. sensory reactivity). To create these dichotomous variables, we used the following cut-off points: total SSP < 155; tactile sensitivity < 30; taste/smell sensitivity < 15; movement sensitivity < 13; under-responsive/seeks sensation < 27; auditory filtering <23; low energy/weak < 26, visual/audiovisual sensitivity < 19.

#### Sleep Duration and Quality

To determine children's sleep duration, we used the following questions: (1) How many hours per day does his son/her daughter sleep on weekdays? and (2) How many hours per day does his son/her daughter sleep during the weekend? The responses to both questions allowed us to obtain the average daily hours that the children slept. Using the recommendations for amount of sleep for pediatric populations of the American Academy of Sleep Medicine (28), children were classified as those who regularly sleep <10 h/day and those regularly sleeping 10 or more hours per day.

To detect those children who had sleep disturbances, we used the adapted and validated short Spanish version of the Pediatric Sleep Questionnaire (PSQ) (29, 30). This version of the PSQ was found to be a suitable tool both for clinical examination and epidemiological research purposes (29). From a clinical perspective, the PSQ can serve as first screening to detect potential sleep disorders as well as to select those children who will require further more detailed medical examination to make an accurate diagnosis. From an epidemiological approach, the PSQ score can be interpreted in terms of group at risk (in this case, children with poor sleep quality) to examine associations that can provide an insight into the determinants and health-related outcomes or events that could be linked to the health problem of interest (in this case, sensory reactivity). This questionnaire is a parent report measure that was specifically designed for identifying sleep-related breathing disorders in children aged from 2 to 18. The PSQ consists of 22 questions grouped into three different sections: (A) questions concerning the children's sleep behavior during the night [9 items]; (B) questions about children's behavior and other possible problems during daytime (e.g., drowsiness, lack of attention, etc.) [7 items]; and (C) questions about ADHD-related behavior according to the DSM-4 criteria [6 items]. Each item from section A and B provides three response options (“yes,” “no,” and “don't know”) that can be scored with the value of 1, 0, or “missing,” respectively. The items from section C can be answered by selecting one of the following response options: never, sometimes, many times, and almost always. To standardize the scoring of all the items, the answers “never” and “sometimes” were categorized as “no” and scored 0 points, and the answers “often” or “almost always” as “yes” and scored 1 point. To obtain the total PSQ score, the total sum of all affirmative responses (“yes”) was calculated and subsequently divided by the total sum of negative (“no”) and affirmative (“yes”) responses. The total score of 0.33 was the cut-off point (29, 30) below which children were categorized as presenting sleep disturbance and was used as a proxy for classifying children according to their sleep quality (i.e., good vs. poor sleep quality).

#### Study Covariates

Parental and child sociodemographic and lifestyle data were collected from the questionnaire reported by the parents. Based on previous solid evidence on the link between socio-demographic and lifestyle factors and child neurodevelopmental outcomes (31–35), and after statistical exploration of our data, we finally selected the following covariates: Parental characteristics, such as age (in years), country of origin (Spain, others), education level (primary or less, secondary, university), employment (yes, no), television watching (<2, ≥2 h/day) and sleep duration (in hours per day); and child characteristics, such as sex (male, female), gestation weeks (<37, ≥37 weeks), birthweight (<2,500, ≥2,500 g), adherence to the Mediterranean diet as measured by the KIDMED index (low, medium, high) (36), parental-reported body mass index (BMI) calculated as weight in kilograms divided by the height in square meters, parental-reported global physical activity (sedentary/less active, active/very active), television watching (<2, ≥2 h/day). Moreover, since clinical or medical conditions may be potential factors that can be related to sensory outcomes, child medical condition (yes, no) was also taken into account. The information on child's health problems was collected asking the parents if their child had been diagnosed with any medical condition or disease. Given that we did not have access to medical records, this question could serve as a proxy for assessing whether a health condition can be related to sensory processing outcomes. Further details on covariates are available elsewhere (24).

### Statistical Analysis

Statistical analyses were carried out using software R, version 4.0.2 (R Foundation for Statistical Computing, Vienna, Austria; http://www.R-project.org). Bilateral statistical tests were applied with a significance level set at 0.05. Normal distribution of the continuous variables was checked using the Kolmogorov-Sminrov test.

General description of the characteristics of the parents and their children by child sensory profile (i.e., SSP < 155 vs. SSP ≥ 155) was performed using frequencies and percentages (%) for the categorical variables, and median and interquartile range (RI) for the continuous variables, given that they were not normally distributed. To evaluate if there were differences in the study characteristics between SSP groups, we applied the Chi-square test (χ^2^) or Fisher's Exact test (categorical variables) and Mann-Whitney's *U*-test (continuous variables).

To assess the association between children's sleep (duration and quality) and prevalence of sensory reactivity, we estimated prevalence ratios (PR) and their respective confidence intervals (CI) at 95% using Poisson multiple regression models with robust variance based on the sandwich estimation of Huber (37, 38). Because of non-convergence, Poisson regression with robust variance was applied instead of the log-binomial regression (39).

To control potential confounding factors, all the models were adjusted for those variables that had shown a *p*-value <0.20 in the bivariate analysis and that produced changes >10% in the association when building the core model. Paternal variables and the child's BMI were not included in the models due to the large number of missing data in these variables, although, they were then allowed for the sensitivity analysis in order to evaluate their possible effect on the main association. Finally, the core model included the following variables: sex, global physical activity, television watching, adherence to a Mediterranean diet with regard to the children's characteristics; and age, education level, country of origin, employment and television watching with respect to the mother's features.

To evaluate the robustness of the findings in which significant associations were observed, we conducted several sensitivity analyses. We performed stratified models to assess the effect of children's sex (boy, *n* = 292; girl, *n* = 287) and age (3–4 years, *n* = 179; 5 years, *n* = 194; 6–7 years, *n* = 206). Moreover, we checked if there were changes in the main effects after the exclusion of the following children's features: sleep duration (<10 h/day; *n* = 189), born preterm (<37 weeks of gestation; *n* = 223), low birthweight (<2,500 g; *n* = 78), medical condition (yes; *n* = 48). Regarding children classified as having sensory reactivity (including both total SSP and each SSP subscale), we also evaluated if there were differences in the effect of main associations between those children with sensory profile characterized as “probable difference” and those as “definite difference.” Finally, several models adjusted for the father's variables such as age, education level, employment and country of origin (*n* = 513) and for the children's BMI (*n* = 460) were also estimated.

## Results

In this study, the prevalence of children with sensory reactivity was 29.5% for total SSP (<155), 11.4% for tactile sensitivity (<30), 15% for taste/smell sensitivity (<15), 22.5% for movement sensitivity (<13), 49.1% for under-responsive/seeks sensation (<27), 44.4% for auditory filtering (<23), 12.4% for low energy/weak (<26), and 25.4% for visual/auditory sensitivity (<19). Regarding children's sleep, 10.4% presented poor sleep quality and around a third (32.6%; *n* = 189) slept <10 h/day.

Table 1 shows the general characteristics of the participants in the InProS study by the children's sensory profile according to whether they presented sensory reactivity (SSP < 155) or not (SSP ≥ 155). Compared to the children who had typical sensory performance, children classified as having sensory reactivity had younger mothers (median age 37 vs. 38), a greater percentage of parents born in a foreign country (mothers = 25.1 vs. 10.3%; fathers = 25.2 vs. 12.0%), a higher proportion of unemployed mothers (40.4 vs. 26.2%) and of fathers with secondary studies (39.5 vs. 30.9%). Regarding children's features, these children had a higher sedentary lifestyle (TV watching ≥ 2 h/day = 64.5 vs. 47.4%) and poor eating habits (i.e., lower median adherence to the Mediterranean diet = 7 vs. 8 points).

**Table 1:** Sociodemographic and lifestyle characterisitics of the parents and their children's sensory profile (SR = SSP < 155) vs. (no SR = SSP ≥155) from the InProS project (*n* = 579).

**Study variables**	**All**	**Sensory profile**^**a**^		**P**^**b**^
		**no SR**	**SR**	
	***n* (%)**	***n* (%)**	***n* (%)**	
	**579 (100)**	**408 (70.5)**	**173 (29.5)**	
**Maternal characteristics**				
Age (years), median (IR)	38 (35–41)	38 (35–41)	37 (33–41)	0.002
Country of birth, *n* (%)				<0.001
Spain	494 (85.3)	366 (89.7)	128 (74.9)	
Others	85 (14.7)	42 (10.3)	43 (25.1)	
Education level, *n* (%)				0.053
Primary or less studies	135 (23.3)	87 (21.3)	48 (28.1)	
Secondary studies	195 (33.7)	133 (32.6)	62 (36.3)	
University studies	249 (43.0)	188 (46.1)	61 (35.7)	
Employment, *n* (%)				0.001
Yes	403 (69.6)	301 (73.8)	102 (59.6)	
No	176 (30.4)	107 (26.2)	69 (40.4)	
TV watching, *n* (%); (missing, *n* = 11)				0.052
<2 h/day	273 (48.1)	204 (50.7)	69 (41.6)	
≥2 h/day	295 (51.9)	198 (49.3)	97 (58.4)	
Sleep duration, median (IR); (missing, *n* = 11)	7.6 (7–8)	7.6 (7–8)	7.6 (7–8)	0.370
**Paternal characteristics**^**c**^				
Age (years), median (IR)	40 (37–43)	40 (37–43)	40 (36–43)	0.159
Country of birth, *n* (%)				<0.001
Spain	432 (84.2)	322 (88)	110 (74.8)	
Other country	81 (15.8)	44 (12)	37 (25.2)	
Education level, *n* (%)				0.006
Primary or less	162 (31.6)	109 (29.8)	53 (36.1)	
Secondary	171 (33.3)	113 (30.9)	58 (39.5)	
University studies	180 (35.1)	144 (39.3)	36 (24.5)	
Employment, *n* (%)				0.528
Yes	458 (89.3)	329 (89.9)	129 (87.8)	
No	55 (10.7)	37 (10.1)	18 (12.2)	
TV watching, *n* (%); (missing, *n* = 28)				0.476
<2 h/day	208 (42.9)	153 (44.0)	55 (40.1)	
≥2 h/day	277 (57.1)	195 (56.0)	82 (59.9)	
Sleep duration, median (IR); (missing, *n* = 28)	7.3 (7–8)	7.3 (7–8)	7.4 (7–8)	0.106
**Child characteristics**				
Sex, *n* (%)				0.002
Male	292 (50.4)	189 (46.3)	103 (60.2)	
Female	287 (49.6)	219 (53.7)	68 (39.8)	
Age (years), median (IR)	5 (4–6)	5 (4–6)	5 (4–6)	0.425
TV watching, *n* (%); (missing, *n* = 12)				<0.001
<2 h/day	270 (47.6)	211 (52.6)	59 (35.5)	
≥2 h/day	297 (52.4)	190 (47.4)	107 (64.5)	
Adherence to MD, median (IR); (missing, *n* = 7)	8 (6–9)	8 (7–9)	7 (6–9)	0.004
Body mass index, median (IR); (missing, *n* = 119)	16 (14.5–17.4)	15.8 (14.4–17.4)	16 (14.9–17.3)	0.188
Physical activity, *n* (%); (missing, *n* = 7)				0.058
Sedentary/poor active	120 (21.0)	76 (18.9)	44 (26.0)	
Active/very active	452 (79.0)	327 (81.1)	125 (74.0)	

*IR, Interquartile range; SR, sensory reactivity; MD, Mediterraean Diet*.

a *Children's sensory profile was determined following the cut-off points proposed by W. Dunn for total SSP score to classify children's level of sensory processing performance as having SR (<155 points) or not having SR (≥155 points)*.

b *P-value was calculated by Chi-square test or Fisher's exact test for categorical variables and by U Mann–Whitney test for continuous variables*.

c *Paternal information is available for 513 parents*.

Table 2 displays the comparison of the children's sleep duration and quality according to the children's sensory profile (sensory reactivity vs. no sensory reactivity), both for the total SSP scoring and for the SSP subscales. In general, sleep duration did not show statistically significant differences in SSP scales. There were practically no differences in the rates of sensory reactivity between those children who slept a median of <10 h a day and those with a median of 10 or more hours a day. On the contrary, it was observed that, compared with those who had good sleep quality, children who presented poor sleep quality had a greater rates of sensory reactivity, both for total SSP as well as for all the SSP subscales, excepting movement sensitivity subscale. However, the percentage of children with poor sleep quality and sensory reactivity was notably higher in the total SSP (66.7%) and under-responsive/seeks sensation (86.7%) and auditory filtering (83.3%) were by far the SSP subscales.

**Table 2:** Comparison of sleep duration and quality^a^ according to sensory processing profile categorized as SR and no SR for total SSP score and each SSP subscale scores in children aged 3-7 from the InProS Project (*n* = 579).

	**All**	**Sleep duration**		***P*-value**^**b**^	**Sleep quality**^**a**^		***P*-value**^**b**^
		**<10 h/d**	**≥10 h/d**		**Poor**	**Good**	
	***n* (%)**	***n* (%)**	***n* (%)**		***n* (%)**	***n* (%)**	
**Sensory processing items**, ***n*** **(%)**	579 (100)	189 (32.6)	390 (67.4)		60 (10.4)	519 (89.6)	
Total SSP (Items 1–38)				0.244			<0.001
no SR (155–190 points)	408 (70.5)	127 (67.2)	281 (72.1)		20 (33.3)	388 (74.8)	
SR (38–154 points)	171 (29.5)	62 (32.8)	109 (27.9)		40 (66.7)	131 (25.2)	
Tactile sensitivity (Ítems 1–7)				1.000			0.049
no SR (30–35 points)	513 (88.6)	168 (88.9)	345 (88.5)		48 (80.0)	465 (89.6)	
SR (7–29 points)	66 (11.4)	21 (11.1)	45 (11.5)		12 (20.0)	54 (10.4)	
Taste/smell sensitivity (Ítems 8–11)				0.063			<0.001
no SR (15–20 points)	492 (85.0)	153 (81.0)	339 (86.9)		40 (66.7)	452 (87.1)	
SR (4–14 points)	87 (15.0)	36 (19.0)	51 (13.1)		20 (33.3)	67 (12.9)	
Movement sensitivity (Ítems 12–14)				0.244			0.870
no SR (13–15 points)	449 (77.5)	141 (74.6)	308 (79.0)		46 (76.7)	403 (77.6)	
SR (3–12 points)	130 (22.5)	48 (25.4)	82 (21.0)		14 (23.3)	116 (22.4)	
Under-responsive/seeks sensation (Ítems 15–21)				0.051			<0.001
no SR (27–35 points)	295 (50.9)	85 (45.0)	210 (53.8)		8 (13.3)	287 (55.3)	
SR (7–26 points)	284 (49.1)	104 (55.0)	180 (46.2)		52 (86.7)	232 (44.7)	
Auditory filtering (Ítems 22–27)				0.154			<0.001
no SR (23–30 points)	322 (55.6)	97 (51.3)	225 (57.7)		10 (16.7)	312 (60.1)	
SR (6–22 points)	257 (44.4)	92 (48.7)	165 (42.3)		50 (83.3)	207 (39.9)	
Low energy/weak (Ítems 28–33)				0.229			0.001
no SR (26–30 points)	507 (87.6)	161 (85.2)	346 (88.7)		44 (73.3)	463 (89.2)	
SR (6–25 points)	72 (12.4)	28 (14.8)	44 (11.3)		16 (26.7)	56 (10.8)	
Visual/auditory sensitivity (Ítems 34–38)				0.223			0.004
no SR (19–25 points)	432 (74.6)	135 (71.4)	297 (76.2)		35 (58.3)	397 (76.5)	
SR (5–18 points)	147 (25.4)	54 (28.6)	93 (23.8)		25 (41.7)	122 (23.5)	

*SSP, Short Sensory Profile; SR, sensory reactivity; h/d, hours per day*.

a *Children's sleep quality was determined by the Pediatric Sleep Questionnaire (PSQ)*.

b *P-value was calculated by Fisher's exact test*.

The association between the children's sleep duration and quality and prevalence of sensory reactivity, both for the total SSP and for the SSP subscales, is shown in Table 3. The results on the relationship between children's sleep duration and prevalence of sensory reactivity disclosed no statistically significant associations. In contrast, with the exception of the results for tactile sensitivity and movement sensitivity, total SSP and the rest of SSP subscales were statistically significantly associated with poor sleep quality, including both the crude analyses (i.e., model 1) and multiple analyses (i.e., model 2).

**Table 3:** Association between sleep duration and quality and prevalence of sensory reactivity for the total SSP score and SSP subscale scores in children aged 3–7 years from InProS project (*n* = 579).

	**SR (%)**	**Children's sleep**							
		**Duration (<10 h/day)**				**Poor quality**			
		***n* cases (%)**	**PR**	**95% CI**	***P*-value**	***n* cases (%)**	**PR**	**95% CI**	***P*-value**
**SHORT SENSORY PROFILE ITEMS**									
Total SSP score (SR < 155 points)	29.5	62 (32.8)				40 (66.7)			
Model 1			0.97	0.94–1.00	0.095		1.33	1.23–0.44	<0.001
Model 2			0.99	0.96–1.02	0.654		1.27	1.18–1.38	<0.001
Tactile sensitivity (SR < 30 points)	11.4	21 (11.1)				12 (20.0)			
Model 1			0.99	0.97–1.02	0.581		1.09	1.00–1.19	0.062
Model 2			1.00	0.98–1.03	0.790		1.05	0.97–1.14	0.239
Taste/smell sensitivity (SR < 15 points)	15.0	36 (19.0)				20 (33.3)			
Model 1			0.97	0.94–1.00	0.026		1.18	1.08–1.30	<0.001
Model 2			0.98	0.96–1.01	0.214		1.15	1.04–1.26	0.005
Movement sensitivity (SR < 13 points)	22.5	48 (25.4)				14 (23.3)			
Model 1			0.96	0.94–0.99	0.007		1.01	0.92–1.10	0.864
Model 2			0.98	0.95–1.00	0.082		0.97	0.88–1.07	0.550
Underresponsive/seek sensation (SR < 26 points)	49.0	104 (55.0)				52 (86.7)			
Model 1			0.97	0.94–1.00	0.050		1.28	1.20–1.37	<0.001
Model 2			0.99	0.96–1.02	0.474		1.21	1.13–1.30	<0.001
Auditory filtering (SR < 23 points)	44.4	92 (48.7)				50 (83.3)			
Model 1			0.99	0.96–1.02	0.406		1.31	1.23–1.39	<0.001
Model 2			1.00	0.97–1.03	0.862		1.26	1.18–1.35	<0.001
Low energy/weak (SR < 26 points)	12.4	28 (14.8)				16 (26.7)			
Model 1			0.99	0.97–1.02	0.583		1.14	1.04–1.25	0.004
Model 2			1.01	0.98–1.03	0.563		1.13	1.04–1.22	0.004
Visual/auditory sensitivity (SR < 19 points)	25.4	54 (28.6)				25 (41.7)			
Model 1			0.99	0.96–1.02	0.334		1.15	1.05–1.26	0.004
Model 2			0.99	0.96–1.02	0.472		1.14	1.04–1.24	0.004

*SR, sensory reactivity; SSP, Short Sensory Profile; PR, Prevalence ratio; CI, Confidence interval. Model 1 is a crude model. Model 2 was adjusted for child's characteristics: sex (female; male), physical activity (sedentary/poor active; active/very active; missing), TV watching (<2 h/day; ≥2 h/day; missing), and adherence to Mediterranean diet (low; medium; high; missing); and for mother's characteristics: age (in years), educational level (primary or less; secondary; university studies), country of birth (Spain; other country), employment (no; yes) and TV watching (<2 h/day; ≥ 2 h/day; missing). Moreover, children's sleep quality (good; poor) was also included in model 2 when assessing the effect of children's sleep duration; and inversely, children's sleep duration (<10 h/day; ≥10 h/day) was added to model 2 when assessing children's sleep quality*.

Sensitivity analyses of the main findings are presented in Table 4. Overall, no substantial changes in the main associations were observed when stratifying analysis by relevant children's characteristics or when adjusting the core model for important paternal variables or for the child's BMI. However, a significant drop in the effects and loss of statistical significance after applying several conditions were observed in the following SSP scales: taste/smell sensitivity, when including only children aged 6–7 (PR = 1,07; 95% CI, 0.91–1.26) and those with probable atypical sensory processing (PR = 1.07; 95% CI, 0.96–1.21); under-responsive/seeks sensation, when including only children aged 3–4; low energy/weak, when including only children aged 5 (PR = 1.01; 95% CI, 0.90–1.13) and 6–7 (PR = 1.04; 95% CI, 0.91–1.19) and after excluding preterm children (PR = 1.03; 95% CI, 0.94–1.14); and visual/auditory sensitivity, when including only girls (PR = 0.96; 95% CI, 0.81–1.13), children aged 3–4 (PR = 1.09; 95% CI, 0.92–1.28), those with probable atypical sensory processing (PR = 0.97; 95% CI, 0.87–1.09).

**Table 4:** Sensitivity analysis of the adjusted prevalence ratios between poor sleep quality and sensory reactivity as measured by the total SSP, taste/smell sensitivity, underresponsive/seek sensation, auditory filtering, low energy/weak, and visual/auditory sensitivity in children aged 3–7 from InPros Project (Alicante, Spain).

	**SR/total cases**	**Sensory reactivity (SR)**								
		**Total SSP score** **(<155 points)**			**Taste/smell sensitivity** **(<15 points)**			**Underresponsive/seek sensation** **(<26 points)**		
		**SR/total cases**^**d**^	**PR (CI 95%)**	***P*-value**	**SR/total cases**^**d**^	**PR (CI 95%)**	***P*-value**	**SR/total cases**^**d**^	**PR (CI 95%)**	***P*-value**
Complete model^a^	171/579	40/60	1.27 (1.18–1.38)	<0.001	20/60	1.15 (1.04–1.26)	0.005	52/60	1.21 (1.13–1.30)	<0.001
Including only boys	103/292	30/41	1.32 (1.20–1.44)	<0.001	13/41	1.12 (1.00–1.25)	0.047	37/41	1.22 (1.13–1.31)	<0.001
Including only girls	68/287	10/19	1.18 (1.01–1.38)	0.042	7/19	1.18 (1.00–1.39)	0.050	15/19	1.23 (1.06–1.43)	0.007
Including only children aged 3–4	57/179	12/19	1.26 (1.10–1.44)	<0.001	7/19	1.18 (1.03–1.36)	0.020	15/19	1.09 (0.95–1.25)	0.225
Including only children aged 5	57/194	12/17	1.33 (1.17–1.51)	<0.001	6/17	1.18 (0.99–1.41)	0.072	16/17	1.26 (1.15–1.39)	<0.001
Including only children aged 6–7	57/206	16/24	1.25 (1.10–1.43)	<0.001	7/24	1.07 (0.91–1.26)	0.402	21/24	1.30 (1.16–1.47)	<0.001
Excluding sleep duration <10 h/day	109/390	23/33	1.33 (1.20–1.47)	<0.001	11/33	1.22 (1.08–1.38)	0.002	27/33	1.20 (1.08–1.33)	<0.001
Excluding preterm children	96/356	19/33	1.22 (1.09–1.37)	<0.001	12/33	1.18 (1.04–1.34)	0.008	27/33	1.16 (1.05–1.29)	0.005
Excluding low weight at birth	148/501	36/55	1.26 (1.16–1.37)	<0.001	19/55	1.15 (1.04–1.27)	0.005	48/55	1.21 (1.13–1.31)	<0.001
Excluding children with some diseases	150/531	27/41	1.26 (1.14–1.38)	<0.001	15/41	1.16 (1.04–1.30)	0.009	35/41	1.22 (1.12–1.32)	<0.001
Adjusted for child body mass index	135/460	31/44	1.29 (1.18–1.41)	<0.001	13/44	1.11 (0.99–1.23)	0.066	39/44	1.24 (1.15–1.33)	<0.001
Adjusted for father's age, education, employed, and country of birth	147/513	34/47	1.32 (1.21–1.43)	<0.001	17/47	1.16 (1.05–1.28)	0.004	43/47	1.27 (1.19–1.37)	<0.001
Including only probable atypical sensory processing^b^	99/507	12/32	1.13 (1.00–1.28)	0.059	6/32	1.07 (0.96–1.21)	0.225	24/32	1.14 (1.02–1.27)	0.024
Including only definitive atypical sensory processing^c^	72/480	28/48	1.36 (1.25–1.49)	<0.001	16/48	1.16 (1.04–1.29)	0.006	40/48	1.23 (1.13–1.34)	<0.001
	**SR/total cases**	**Auditory filtering** **(**<**23 points)**			**Low energy/weak** **(**<**26 points)**			**Visual/auditory sensitivity** **(**<**19 points)**		
		**SR/total cases**^**d**^	**PR (CI 95%)**	***P*****-value**	**SR/total cases**^**d**^	**PR (CI 95%)**	***P*****-value**	**SR/total cases**^**d**^	**PR (CI 95%)**	***P*****-value**
Complete model^a^	171/579	50/60	1.26 (1.18–1.35)	<0.001	16/60	1.13 (1.04–1.22)	0.004	25/60	1.14 (1.04–1.24)	0.004
Including only boys	103/292	34/41	1.22 (1.13–1.32)	<0.001	9/41	1.09 (0.99–1.20)	0.069	21/41	1.25 (1.13–1.38)	<0.001
Including only girls	68/287	16/19	1.31 (1.17–1.46)	<0.001	7/19	1.20 (1.04–1.39)	0.013	4/19	0.96 (0.81–1.13)	0.605
Including only children aged 3–4	57/179	16/19	1.23 (1.09–1.38)	<0.001	9/19	1.29 (1.12–1.48)	<0.001	8/19	1.09 (0.92–1.28)	0.325
Including only children aged 5	57/194	14/17	1.35 (1.20–1.52)	<0.001	2/17	1.01 (0.90–1.13)	0.894	7/17	1.20 (1.02–1.42)	0.029
Including only children aged 6–7	57/206	20/24	1.22 (1.09–1.36)	<0.001	5/24	1.04 (0.91–1.19)	0.553	10/24	1.13 (0.98–1.30)	0.082
Excluding sleep duration <10 h/day	109/390	29/33	1.31 (1.21–1.42)	<0.001	10/33	1.16 (1.03–1.31)	0.015	11/33	1.10 (0.97–1.25)	0.150
Excluding preterm children	96/356	26/33	1.24 (1.13–1.36)	<0.001	5/33	1.03 (0.94–1.14)	0.503	13/33	1.12 (1.00–1.25)	0.043
Excluding low weight at birth	148/501	46/55	1.27 (1.19–1.36)	<0.001	14/55	1.11 (1.02–1.21)	0.020	22/55	1.13 (1.03–1.24)	0.011
Excluding children with some diseases	150/531	32/41	1.23 (1.13–1.34)	<0.001	12/41	1.12 (1.02–1.23)	0.016	20/41	1.18 (1.06–1.30)	0.002
Adjusted for child body mass index	135/460	35/44	1.28 (1.18–1.38)	<0.001	11/44	1.12 (1.02–1.23)	0.021	22/44	1.19 (1.07–1.31)	<0.001
Adjusted for father's age, education, employed and country of birth	147/513	39/47	1.29 (1.20–1.38)	<0.001	11/47	1.09 (1.00–1.19)	0.059	21/47	1.15 (1.04–1.26)	0.006
Including only children with probable difference^b^	99/507	23/32	1.23 (1.12–1.36)	<0.001	2/32	1.01 (0.95–1.08)	0.714	4/32	0.97 (0.87–1.09)	0.613
Including only children with definitive difference^c^	72/480	40/48	1.34 (1.24–1.44)	<0.001	33/48	1.18 (1.07–1.29)	<0.001	16/48	1.23 (1.12–1.35)	<0.001

*SSP, Short Sensory Profile; SR, sensory reactivity*.

a *Complete model was the model 2 used in Table 3*.

b *Children with probable difference as measured by the SSP were classified as follows: Total SSP (<142), taste/smell sensitivity (<12), under-responsive/seeks sensation (<24), auditory filtering (<20), low energy/weak (<24), and visual/auditory sensitivity (<16). These children were compared with their respective peers classified as having typical sensory performance (i.e., ≥155, ≥15, ≥27, ≥23, ≥26, ≥19, respectively)*.

c *Children with definite difference as measured by the SSP were classified as follows: Total SSP (≤141), taste/smell sensitivity (≤11), under-responsive/seeks sensation (≤23), auditory filtering (≤19), low energy/weak (≤23), and visual/auditory sensitivity (≤15). These children were compared with their respective peers classified as having typical sensory performance (i.e., ≥155, ≥15, ≥27, ≥23, ≥26, ≥19, respectively)*.

d *Frequency of children with sensory reactivity (SR) and poor sleep quality*.

## Discussion

Main findings of this study showed that poor sleep quality in children aged 3–7 was significantly associated with greater prevalence of sensory reactivity as measured by total SSP, tactile sensitivity, taste/smell sensitivity, under-responsive/seeks sensation, auditory filtering, low energy/weak and visual/auditory sensitivity SSP subscales. However, no significant associations were observed between sleep duration and prevalence of sensory reactivity in children at this age range. To the best of our knowledge, this is the first time this association has been reported and explored in a population-based sample of school-age children.

Previous research has clearly documented that sensory processing issues and sleep disturbances are common symptoms in children with developmental disabilities such as ASD (40). However, the available evidence about the relationship between the children's sensory processing issues and sleep outcomes is very limited and based on preliminary results mainly obtained from descriptive analyses (18–23), which indicates that this health issue is still in its early stages of research. In this regard, it should be noted that an important point of marked difference between the present study and previous research lies in the fact that we applied an evident epidemiological approach to investigate this health issue. As such, to improve the understanding of and provide better consistency with the suggested relationship between children's sleep and sensory processing, we quantified the strength of this association using adequate measures of the effect and determined its extent accounting for potential confounding factors. Notwithstanding that the epidemiological design of this study could prevent to some extent a direct comparison of our results with those from other previous studies, our approach should be understood as an important step forward in establishing a more clearly defined research pathway of this health issue. From this perspective, our results help to confirm research findings of prior studies, thus adding convincing evidence for suggesting a likely link between poor sleep quality and sensory reactivity during childhood.

Although, the cross-sectional design of this study does not allow inferring causality, there is preliminary evidence from neuroscientific research suggesting that sleep and sensory processing may be causally and reciprocally related *via* neurophysiological mechanisms (41–43). Hence, the potential interplay between sleep and sensory processing functioning could partly explain why we observed a global effect on sensory processing outcomes as indicated by total SSP and almost all SSP subscales. From this standpoint, our results should be interpreted as a significant extension of initial evidence not only by the fact that these findings confirm that sleep-related problems are likely connected with sensory reactivity (18–23), and more specifically, with some specific sensory functions such as auditory filtering (23–25) and tactile sensitivity (20), but also by the fact that they quantified this relationship in epidemiological terms. Moreover, to evaluate the strength of the effect of the main findings, we did test with sensitivity analyses whether sensory reactivity could be related in part to other potential parental or child's characteristics. When applying the models based on a set of assumptions, the results remained largely similar to those shown for the main analyses. However, as displayed, child's age and sex and being preterm are factors that could play a role in sensory reactivity, but we must also consider that most of these estimates were based on low numbers. Although, pending additional confirmation from further research on how and to what extent sleep and sensory processing may be related, it can be postulated that children with poor sleep quality may be more likely to display sensory reactivity, thereby failing to selectively regulate their sensitivity to sensory stimuli.

In the absence of a clear answer about the neural mechanisms whereby sleep and sensory processing may be connected (41–43), a plausible explanation for the potential relationship between poor children's sleep quality and sensory reactivity could initially lie in the fact that both factors have been separately associated with higher cortisol levels. Several studies observed that children with sensory processing sensitivity —hyper/over sensitivity—, who tend to be easily overwhelmed and agitated by stressors and adversity (1, 44), displayed increased physiological stress responses to sensory stimuli as indicated by elevated cortisol levels (45–47). Similarly, there is some evidence suggesting that poor sleep quality is associated with virtually the same behavioral effects and exaggerated cortisol stress responses in child populations (48–50). Thus, in line with that mentioned earlier, it would seem reasonable to propose an interesting working hypothesis on the basis that both impaired functions can share common neural pathways displaying equivalent neuroendocrine and psychological responses. Following this line of research, early results indicated that alterations in the autonomic nervous system (ANS) and the hypothalamic-pituitary-adrenal (HPA) axis seem to play an important role in sensory processing functioning and sleep, hence, it is assumed that they could be responsible for sensory reactivity (44, 46, 51) as well as sleep disturbances (49) in childhood. Interestingly, a recent neural research study produced in parallel promising results suggesting that sensory processing and sleep may be linked *via* thalamic circuit mechanisms (41).

Contrary to that observed for sleep quality, we did not find statistically significant associations between sleep duration and prevalence of sensory reactivity in children aged 3–7. Although, consistent with prior findings (19, 21), this could be partly attributed to the fact that sleep duration was very homogenous overall; the daily average of hours of sleep was remarkably similar in children classified as those having or those not having sensory reactivity (9.8 and 9.9 h/day, respectively). Indeed, only five children included in this study slept an average of seven or less hours per day, thus preventing us from assessing the effect of a shorter sleep duration on the prevalence of sensory reactivity. Nevertheless, although, the lack of association between sleep duration and negative sensory processing outcomes can be considered as favorable, we are aware that these results must be interpreted with caution.

This study presents some limitations and strengths that should be taken into account. First, as mentioned above, the cross-sectional design of our study prevents us from establishing a cause-effect relationship from the results we obtained. However, it should be noted that the pioneering nature of our study does offer a comprehensive and accurate description of sensory reactivity in children from the general population using descriptive and analytical epidemiological methods of research that allows us to identify potentially relevant determinants of children's health and development. Moreover, the results of this study can serve as a basis for more detailed research provided by future longitudinal epidemiological studies that will contribute to a better understanding of sensory processing disturbances in childhood. Another significant advantage of this study arises from the fact that the study sample was randomly selected and recruited from the general population. Although, it could be argued that the sample size was not sufficiently large (response rate of 37%), the estimation of study sample size preserved to some extent the degree of representativeness (24). However, we are aware that the results obtained in this study must be corroborated by further high-quality studies with larger samples. In addition, one important limitation of this study that should be acknowledged is that all the information collected from the participants were parent-reported, thus potentially resulting in some misclassification. Nevertheless, if there were any inaccuracy in reporting, it should be considered as non-differential. To ensure the accuracy of the research data and control the possibility of bias, we used valid and reliable instruments employed in previous research studies (4, 5, 52, 53). Although, it may be argued that the PSQ is not the most suitable tool to assess sleep quality in children, because this questionnaire was specifically designed for detecting sleep-related breathing disorders, it is found to be a suitable tool for epidemiological research and can be useful for classifying children at risk of sleep disturbance. In this respect, the PSQ allowed us to identify children as a group at risk (i.e., children with sleep disturbances) to examine associations with sensory reactivity. Thus, in terms of research, the PSQ score can be seen as a good proxy for assessing sleep quality in children. Furthermore, we performed multiple statistical models adjusted for potential confounding factors to analyze data, although, the likelihood of residual confounding or bias due to missing information cannot be disregarded. Finally, we also checked the robustness of our results by conducting different sensitivity analyses to examine the effect of specific conditions that could be related to the children's sensory processing.

## Conclusion

In this population-based study with children aged from 3 to 7, we observed that poor sleep quality was statistically significantly associated with a higher prevalence of sensory reactivity as measured by the total SSP and almost all SSP subscales. To our knowledge, this is the first time that this association has been explored and reported. Although, we did not find any significant association between sleep duration and sensory reactivity, examining the relationship between sleep restriction and sensory processing outcomes should be clearly warranted because of their possible detrimental effects on children's well-being and health. Pending further research from prospective studies, our findings are consistent with early results of the potential link between sleep and sensory processing functioning and add convincing evidence on the basis of an epidemiological approach. Meanwhile, interventions specifically aimed at improving sleep disturbances or sensory processing issues in children should consider including the interplay of both factors as a potential negative determinant that may adversely affect children's health and normal development.

## Data Availability Statement

The raw data supporting the conclusions of this article will be made available by the authors, without undue reservation.

## Ethics Statement

The studies involving human participants were reviewed and approved by Ethics Committee of Miguel Hernandez University of Elche (protocol code DPC.ASP.02.16 approved on 20th December 2016). Written informed consent to participate in this study was provided by the participants' legal guardian/next of kin.

## Author Contributions

E-MN-M and DV-G: conceptualization, methodology, formal analysis, and visualization. E-MN-M: resources, data curation, and supervision. E-MN-M, PF-P, and DV-G: writing—original draft preparation and project administration. PF-P, DV-G, E-MN-M, M-PR-C, CE-S, PP-G, IJ-L, AS-P, IC-S, M-TP-V, and MH-P: writing—review and editing. All authors have read and agree to the published version of the manuscript.

## Conflict of Interest

The authors declare that the research was conducted in the absence of any commercial or financial relationships that could be construed as a potential conflict of interest.
